# Physiological and Redox Responses of Siamese Crocodiles (*Crocodylus siamensis* Schneider, 1801) to Progressive Thermal Exposure

**DOI:** 10.3390/antiox15101309

**Published:** 2026-10-11

**Authors:** Prayuth Kusolrat, Jittraporn Prakaotan, Thitipat Boonsit, Pornnipa Chanpakdee, Mattanawan Jiranuchaiwattana, Worapol Aengwanich

**Affiliations:** 1 Faculty of Science and Technology, Nakhonratchasima Rajabhat University, Nakhonratchasima 30000, Thailand; prayuth.k@nrru.ac.th; 2 Faculty of Veterinary Sciences, Mahasarakham University, Mahasarakham 44000, Thailand; 63012210001@msu.ac.th (J.P.); 63012210009@msu.ac.th (T.B.); 63012210016@msu.ac.th (P.C.); 63012210042@msu.ac.th (M.J.); 3 Stress and Oxidative Stress in Animal Research Unit, Mahasarakham University, Mahasarakham 44000, Thailand

**Keywords:** thermal stress, ectotherm, oxidative stress, antioxidant defense, glutathione peroxidase, crocodilian physiology

## Abstract

Acute temperature elevation can challenge physiological regulation and cellular redox homeostasis in ectotherms, yet integrated physiological and redox responses remain poorly characterized in the Siamese crocodile (*Crocodylus siamensis* Schneider, 1801). This study aimed to evaluate physiological responses during acute warming and characterize biochemical responses in pooled blood cells. Six crocodiles were exposed to temperatures of 30–38 °C, while pooled blood cells were incubated at 30–40 °C. Body temperature increased from 31.35 ± 0.15 °C at 30 °C to 37.48 ± 0.20 °C at 38 °C (*p* < 0.0001), while pulse rate increased from 42.50 ± 5.82 bpm at 34 °C to 62.38 ± 6.33 bpm at 36 °C (*p* < 0.05); blood oxygen saturation remained unchanged. Within the pooled preparation, hydrogen peroxide increased and glutathione peroxidase activity decreased at higher temperatures (*p* < 0.05), accompanied by alterations in ferric iron, lipid hydroperoxide, and malondialdehyde. Mitochondrial activity, total antioxidant capacity, nitric oxide, and protein oxidation remained unchanged. Because the biochemical experiment comprised one pooled biological preparation, these results represent within-preparation responses rather than population-level effects. These findings provide an integrated characterization of whole-animal physiological responses and complementary changes in redox balance, iron status, and lipid peroxidation during acute warming in Siamese crocodiles.

## 1. Introduction

Rising environmental temperatures driven by climate change can affect organisms and ecosystems through changes in physiology, behavior, morphology, and species distribution, with consequences for biological performance and ecological processes [1,2]. Ectothermic animals are particularly sensitive to environmental temperature because their body temperature and many physiological processes are strongly influenced by external thermal conditions, making their physiological performance closely dependent on the surrounding thermal environment [3,4]. As environmental temperature increases, metabolic demand may also rise, increasing the energetic and oxygen requirements needed to maintain physiological function [2,4,5]. When these demands exceed the capacity of physiological compensatory mechanisms, maintaining homeostasis may become increasingly difficult, particularly as thermal conditions approach or exceed the limits of physiological adjustment [2,6].

At the cellular level, elevated temperature may alter metabolic activity and energetic demand, requiring adjustments in cellular processes involved in energy production and utilization [3,5]. Temperature can also influence mitochondrial function and respiratory processes, including components of the electron transport system that are central to cellular energy metabolism [2,7]. When reactive oxygen species exceed antioxidant capacity, redox imbalance can promote oxidative damage and enhance lipid peroxidation, especially within membranes containing high levels of polyunsaturated fatty acids [8,9,10]. Within this process, hydrogen peroxide contributes to redox regulation [11,12], whereas malondialdehyde serves as an indicator of lipid peroxidation [12,13].

Iron redox homeostasis is closely linked to oxidative balance because changes in iron availability and redox state can influence cellular oxidative processes. Through Fenton chemistry, hydrogen peroxide oxidizes ferrous iron, leading to the formation of hydroxyl radicals that contribute to lipid peroxidation and oxidative damage [14]. Disruption of iron redox balance, together with impairment of the glutathione–glutathione peroxidase antioxidant system, can promote lipid peroxide accumulation and compromise cellular antioxidant protection [15]. These alterations are features associated with ferroptosis, a regulated form of cell death driven by iron-dependent processes and linked to disturbances in iron metabolism, antioxidant defenses, and the accumulation of lipid peroxides [14,15,16].

**Figure 1:**
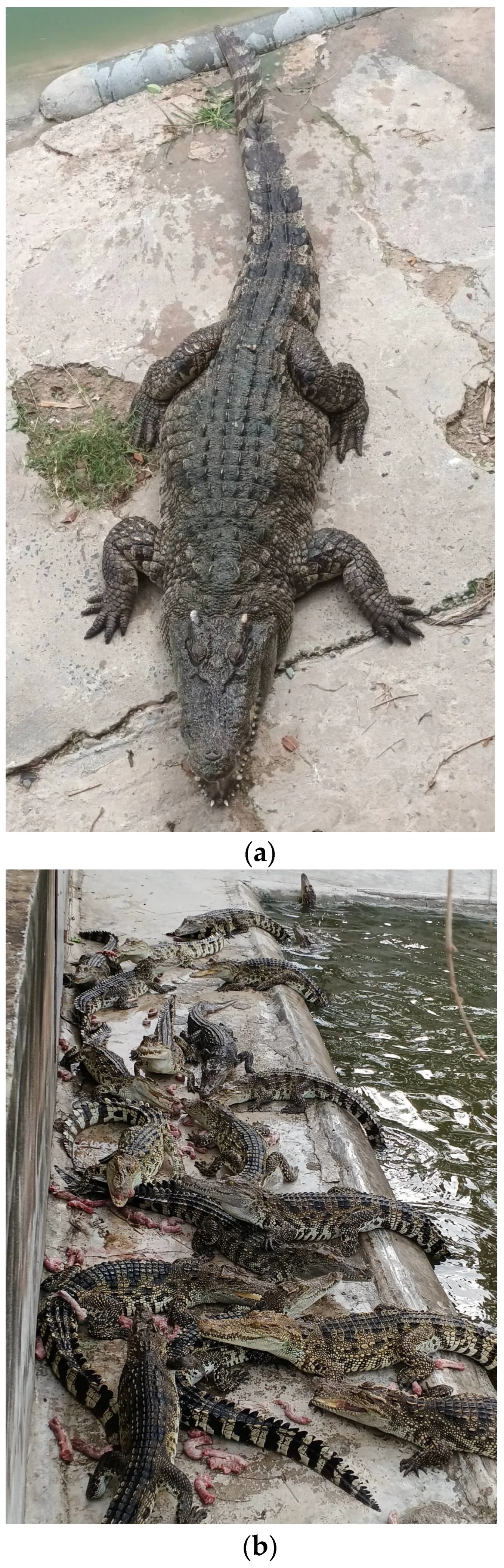
The Siamese crocodile (*Crocodylus siamensis* Schneider, 1801), identified based on morphological characteristics (**a**) and farm records (**b**).

The Siamese crocodile (*Crocodylus siamensis* Schneider, 1801) (Figure 1) is native to Southeast Asia and occupies diverse freshwater habitats [17,18]. The species has ecological importance in freshwater ecosystems [18] and economic value through captive breeding and crocodile-related industries, making it relevant from both conservation and production perspectives [19]. Wild populations remain critically endangered because of habitat loss, hunting, wildlife trade, fishing-related mortality, and other anthropogenic pressures that continue to threaten natural populations [17,18]. Commercial farming is well established in Southeast Asia, particularly in Thailand, Cambodia, and Vietnam, where captive populations support production and may also contribute to conservation through captive-breeding and reintroduction programs [20,21].

Crocodilians regulate body temperature largely through behavioral thermoregulation, including basking and movement between aquatic and terrestrial environments, which allows them to modify heat exchange with their surroundings [22,23]. Elevated environmental temperatures can influence metabolic activity, oxidative balance, and cellular function, indicating that thermal conditions can affect physiological processes at both organismal and cellular levels [24]. Maintaining suitable thermal conditions is therefore relevant to physiological stability and welfare in captive crocodilians and is important for supporting normal physiological function [2,5,23].

Despite the biological and practical importance of temperature regulation in crocodilians, information on the acute thermal responses of Siamese crocodiles remains limited, particularly regarding the relationship between whole-animal physiological responses and cellular biochemical changes during short-term temperature elevation. Previous research has demonstrated antioxidant and anti-inflammatory properties of blood-derived components and hemoglobin hydrolysates from Siamese crocodiles [25,26], while crocodile oil has also been studied for its composition, functional properties, and potential applications [27]. Crocodilian heart rate has been shown to change during heating and cooling [28], but integrated information linking short-term physiological responses with cellular redox regulation during acute temperature elevation remains scarce. Nucleated blood cells of non-mammalian vertebrates retain functional cellular machinery involved in metabolic and redox processes and can serve as minimally invasive cellular proxies for investigating physiological responses, providing an accessible system for examining cellular responses under controlled conditions [29]. Studies in fish, amphibians, and birds have also reported temperature-associated changes in oxidative markers, mitochondrial activity, or blood-cell viability [30,31,32]. However, comparable information on oxidative balance, antioxidant defenses, iron redox status, and lipid peroxidation in Siamese crocodile blood cells remains limited, particularly in relation to acute temperature elevation.

Accordingly, two complementary hypotheses were proposed. For the in vivo experiment, we hypothesized that progressive acute warming would alter physiological responses in Siamese crocodiles, including body temperature, surface temperature, and cardiovascular responses. For the in vitro experiment, we hypothesized that acute temperature elevation would alter indices related to reactive oxygen species, antioxidant defenses, iron redox status, and lipid peroxidation within a standardized pooled blood-cell preparation. Therefore, this study aimed to evaluate physiological responses during progressive acute warming in Siamese crocodiles and to characterize temperature-dependent biochemical responses in pooled Siamese crocodile blood cells under controlled in vitro conditions. Together, these complementary experiments were designed to provide baseline information on physiological responses at the whole-animal level and cellular biochemical responses to acute temperature elevation in this species.

## 2. Materials and Methods

### 2.1. Animals

This study was conducted between May and June 2026 using six clinically healthy Siamese crocodiles (*Crocodylus siamensis* Schneider, 1801), comprising three males and three females. The crocodiles were approximately 18 months old, with a body length of 75 ± 4.27 cm and a body weight of 1.07 ± 0.33 kg (mean ± SD). Each crocodile was individually weighed and measured before the start of the experiment. All crocodiles were obtained from and maintained at a commercial crocodile farm in Roi Et Province, Northeastern Thailand. The sample size of six animals was determined for the physiological repeated-measures experiment using the resource equation method [33], together with consideration of the Replacement, Reduction, and Refinement (3Rs) principles to maintain scientific adequacy while minimizing unnecessary animal use [34]. The study was designed to characterize acute thermal responses in the experimental group rather than to evaluate sex-specific differences.

All crocodiles were examined by veterinarians and confirmed to be clinically healthy. Only crocodiles confirmed to be clinically healthy by veterinary examination and those that had received no medication for at least one month before the study were included. No additional exclusion criteria were applied. The six crocodiles were housed together in a single 5 × 5 m concrete pond with walls 1.5 m high. The pond consisted of a concrete land area for resting and feeding and a 2 × 3 m water area with a depth of 50 cm. Before the experiment, the crocodiles underwent a 14-day acclimatization period. During this period, which occurred during the rainy season in Thailand, the crocodiles were maintained under routine farm conditions without artificial control of air or pond-water temperature; these temperatures were not recorded. They were also accustomed to handling, mouth restraint using an elastic band, transportation, and the monitoring procedures applied during the experiment, including the recording of pulse rate and blood oxygen saturation and the measurement of body and regional surface temperatures. This habituation procedure was used to minimize potential handling-related stress during the experiment.

The crocodiles were fed fresh ground chicken necks and bones at approximately 5–10% of body weight per day, following routine farm husbandry practice. The amount of feed offered was estimated rather than calculated from daily individual body-weight measurements because frequent handling and weighing of crocodiles are impractical and pose safety concerns. Food was withheld for 48 h before the in vivo thermal-exposure experiment, consistent with the fasting period previously used before physiological assessment and blood collection in crocodilians [35].

### 2.2. Experimental Design

Two complementary experiments were performed. The in vivo experiment examined physiological responses of Siamese crocodiles during progressive acute warming, whereas the in vitro experiment examined biochemical responses of pooled blood cells to controlled temperature exposure.

Sub-experiment 1: Physiological responses of Siamese crocodiles to progressive acute warming.

A repeated-measures design was used, with all six crocodiles exposed sequentially to water temperatures of 30, 32, 34, 36, and 38 °C. The animals remained at each target temperature for 30 min before physiological measurements were obtained. The maximum temperature was limited to 38 °C because preliminary observations indicated signs of fatigue during acute exposure at this temperature. Higher temperatures were therefore avoided to prevent unnecessary physiological distress and protect animal welfare. The maximum exposure temperature of 38 °C was therefore used as a welfare-based limit. Animals were monitored throughout the thermal-exposure procedure for signs of excessive distress or fatigue. According to the humane endpoint criteria specified in the IACUC-approved protocol, severe abnormalities in behavior or physical appearance were considered criteria for early termination of the experiment. No adverse events occurred, no animals met the humane endpoint criteria, and no animals required removal from the experiment.

The six crocodiles were assigned to two foam boxes (56 × 40 × 26 cm), with three animals in each box. Water for temperature adjustment was prepared in a water bath (Model WNB45; Memmert GmbH + Co. KG, Schwabach, Germany; 60 × 40 × 23 cm). Both boxes initially contained water at 30 °C. For each subsequent temperature, warm water was gradually added and mixed over approximately 2 min to increase the temperature by 2 °C, corresponding to a heating rate of approximately 1 °C/min.

For handling safety, each crocodile’s snout was secured with an elastic band using the same restraint procedure throughout the experiment. During exposure, the body was immersed while the head, eyes, and nostrils remained above the water surface, approximating the normal aquatic posture of crocodiles. The animals could move freely within the boxes. Physiological measurements included pulse rate, blood oxygen saturation, body temperature, and surface temperatures of the nose, head, back, flank, thigh, tail base, abdomen, and claw. Because the temperature treatments followed a fixed ascending sequence, the effects of temperature could not be separated from those of cumulative exposure time and treatment order. This design feature was therefore considered when interpreting the physiological findings. The study was not blinded. The investigators conducting the experiment were aware of the experimental design, temperature sequence, and experimental conditions, as these procedures were predefined in the research protocol. Data analysis was performed separately from the experimental procedures, and the temperature groups were identified during the analysis.

Sub-experiment 2: Biochemical responses of Siamese crocodile blood cells to acute temperature elevation.

After the in vivo experiment, the crocodiles resumed routine farm conditions for 14 days before blood collection to minimize potential carry-over effects from the preceding thermal exposure. Blood was subsequently obtained from each crocodile via the ventral coccygeal vein. A volume of 1 mL was collected using a 22-gauge, 1.5-inch needle, following the procedure described by Wilhite and Nevarez [36].

Blood collected from the six crocodiles was combined in equal proportions and thoroughly mixed. The resulting blood cells underwent two washing cycles in phosphate-buffered saline (PBS; pH 7.4), with each cycle involving centrifugation at 2500 rpm for 5 min (Hettich Rotina 380R, Hettich GmbH & Co. KG, Tuttlingen, Germany). After washing, the pooled blood-cell preparation was diluted 1:200 with PBS (pH 7.4), thoroughly mixed, and assigned to six temperature treatments: 30, 32, 34, 36, 38, and 40 °C.

The in vitro temperature range was extended to 40 °C to examine cellular responses beyond the maximum temperature used in live animals without exposing the crocodiles to further thermal challenge. At each temperature, five 10 mL aliquots of the diluted pooled preparation were incubated for 20 min in a temperature-controlled water bath. The 20 min incubation period was used as a standardized short duration of cellular exposure applied consistently across all temperature treatments. This duration was not intended to reproduce the duration of thermal exposure in the whole animal or to represent a biologically equivalent exposure period in intact Siamese crocodiles. Therefore, direct extrapolation between the in vitro and in vivo exposure durations is not warranted. The pooled preparation represented one biological experimental unit, while the five aliquots at each temperature were technical replicates used to characterize temperature-dependent variation within the preparation.

After incubation, each sample was centrifuged at 2500 rpm for 5 min at 4 °C, yielding separate supernatant and blood-cell pellet fractions. The supernatant was used to determine reactive oxygen species (hydrogen peroxide and hydroxyl radical), antioxidant-related indices (total antioxidant capacity, glutathione, glutathione peroxidase activity, vitamin E, and cysteine), iron redox status (ferrous and ferric iron), oxidative damage (lipid hydroperoxide, malondialdehyde, and protein oxidation), and nitric oxide. Mitochondrial activity was assessed in the blood-cell pellet.

### 2.3. Determination of Indicators

#### 2.3.1. Physiological Parameters

A pulse oximeter (VE-H100B, Edan, Shenzhen, China) was used to determine pulse rate and blood oxygen saturation, with its sensor positioned on the interdigital webbing of each Siamese crocodile’s right hind limb. Body temperature was determined using a digital thermometer (Model PT-03, PHENOMA Medical, Thailand; manufactured in Shenzhen, China). The probe was introduced approximately 1 cm into the cloaca and maintained in position for 1 min before the temperature was recorded. Surface temperature was measured at eight anatomical sites—the nose, head, back, flank, thigh, tail base, abdomen, and claw—using an infrared thermometer (Wintact^®^, WT323C, Shenzhen, China). Measurements were taken with the infrared thermometer positioned approximately 5 cm from the body surface.

#### 2.3.2. Biochemical Parameters

Mitochondrial activity, hydrogen peroxide, nitric oxide, total antioxidant capacity, glutathione, glutathione peroxidase activity, vitamin E, ferrous iron, ferric iron, malondialdehyde, and protein oxidation were determined using previously described methods [37]. Hydroxyl radical, cysteine, and lipid hydroperoxide were determined as described below.

##### Hydroxyl Radical

Hydroxyl radical formation was quantified using a modified salicylate hydroxylation assay [38]. Briefly, 100 µL of phosphate-buffered saline (pH 7.4), 40 µL of 10 mM sodium salicylate (KemAus, Sydney, Australia), 20 µL of 10 mM ferrous sulfate (Loba Chemie Pvt. Ltd., Mumbai, India), and 40 µL of sample were combined. The reaction mixture was incubated for 10 min at room temperature. Subsequently, 100 µL of ferric iron–acetic acid working reagent was added for color development, and the mixture was allowed to stand for 5–10 min before absorbance was measured at 510–520 nm using a microplate reader (Tecan Trading AG, Männedorf, Switzerland). A standard curve was prepared using hydrogen peroxide (QRëC™, Auckland, New Zealand) at concentrations of 0–5.00 mM. Sample values were calculated from the standard curve and expressed as hydrogen peroxide equivalents.

##### Cysteine Assay

Cysteine was quantified according to the modified Ellman method [39]. A 20 µL sample was combined with 180 µL of phosphate buffer (pH 7.4) and 20 µL of 1 mM 5,5-dithiobis(2-nitrobenzoic acid) (DTNB) (Sigma-Aldrich, Saint Louis, MO, USA; Made in Buchs, Switzerland). The mixture was protected from light and incubated at room temperature for 3–5 min before absorbance was measured at 412 nm using the microplate reader. L-cysteine (Himedia™, Thane, India) served as the standard.

##### Lipid Hydroperoxide

Lipid hydroperoxide was quantified using the modified ferrous oxidation–xylenol orange (FOX) assay of Nourooz-Zadeh et al. [40]. Two 200 µL aliquots of each sample were prepared; one was treated with 10 mM triphenylphosphine (TPP; Glentham Life Sciences, Corsham, UK), whereas the other remained untreated. Both aliquots were incubated at room temperature for 30 min. Subsequently, 2000 µL of FOX reagent was added to each aliquot. The reagent contained 90% methanol (QRëC™, Auckland, New Zealand), 25 mM sulfuric acid (Loba Chemie Pvt. Ltd., Mumbai, India), 4 mM butylated hydroxytoluene (BHT) (Loba Chemie Pvt. Ltd., Mumbai, India), 250 µM ferrous ammonium sulfate hexahydrate (Loba Chemie Pvt. Ltd., Mumbai, India), and 100 µM xylenol orange (QRëC™, Auckland, New Zealand). After a further 10 min, absorbance was measured at 560 nm using a microplate reader, with hydrogen peroxide used to generate the standard curve. The untreated aliquot represented the total peroxide-associated signal, whereas TPP treatment reduced the lipid hydroperoxide fraction. Lipid hydroperoxide (LOOH) was calculated as the difference between the untreated and TPP-treated measurements (LOOH = no-TPP − TPP) and quantified using the hydrogen peroxide standard curve.

### 2.4. Statistical Analysis

Normality of the data was examined using the Shapiro–Wilk test [41]. For the physiological measurements, repeated observations from the same crocodiles were evaluated using a mixed-effects model, with temperature specified as the fixed effect and crocodile identity as the random effect. No animals, experimental units, or data points were excluded from the analyses. The biochemical measurements were evaluated using one-way ANOVA, followed by Sidak’s multiple comparisons test for post hoc comparisons. In the in vitro experiment, blood collected from all six crocodiles was combined before temperature exposure; consequently, the resulting pooled blood-cell preparation represented one biological experimental unit. The five aliquots examined at each temperature were technical replicates rather than independent biological replicates. Technical replicate values were not averaged before statistical analysis; individual aliquot measurements were used for comparisons within the pooled preparation. Accordingly, statistical differences in the biochemical measurements describe variation among temperature treatments within this pooled preparation and do not provide animal-level or population-level inference. All analyses were conducted using GraphPad Prism version 11 (GraphPad Software, Boston, MA, USA). Results are expressed as mean ± SEM, with statistical significance defined as *p* < 0.05.

## 3. Results

Physiological responses to progressive acute warming are presented in Table 1. Body and surface temperatures increased with warming, while pulse rate differed significantly between 34 °C and 36 °C (*p* < 0.05); blood oxygen saturation remained unchanged across temperatures (*p* > 0.05).

**Figure 2:**
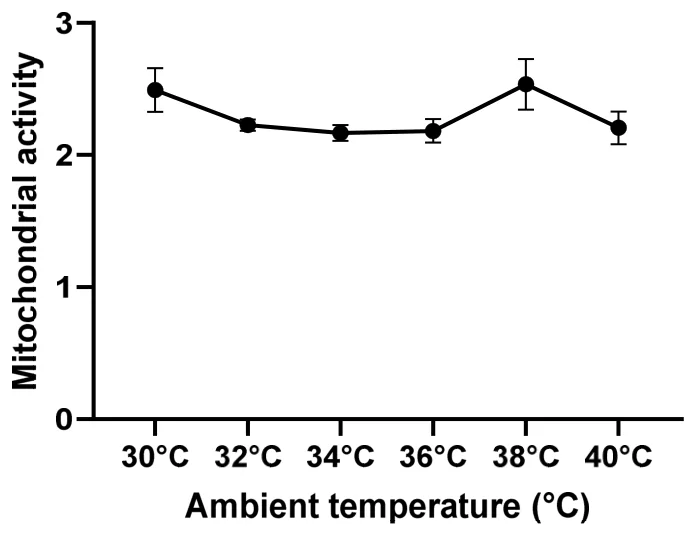
Mitochondrial activity of Siamese crocodile blood cells. Blood from six crocodiles was pooled before temperature exposure to constitute one biological experimental unit. Five technical replicate aliquots were incubated for 20 min at each temperature (30, 32, 34, 36, 38, and 40 °C). Data are presented as mean ± SEM. Statistical comparisons describe temperature-associated variation among technical replicates within the single pooled preparation and do not represent biological replication or inference at the animal or population level.

**Figure 3:**
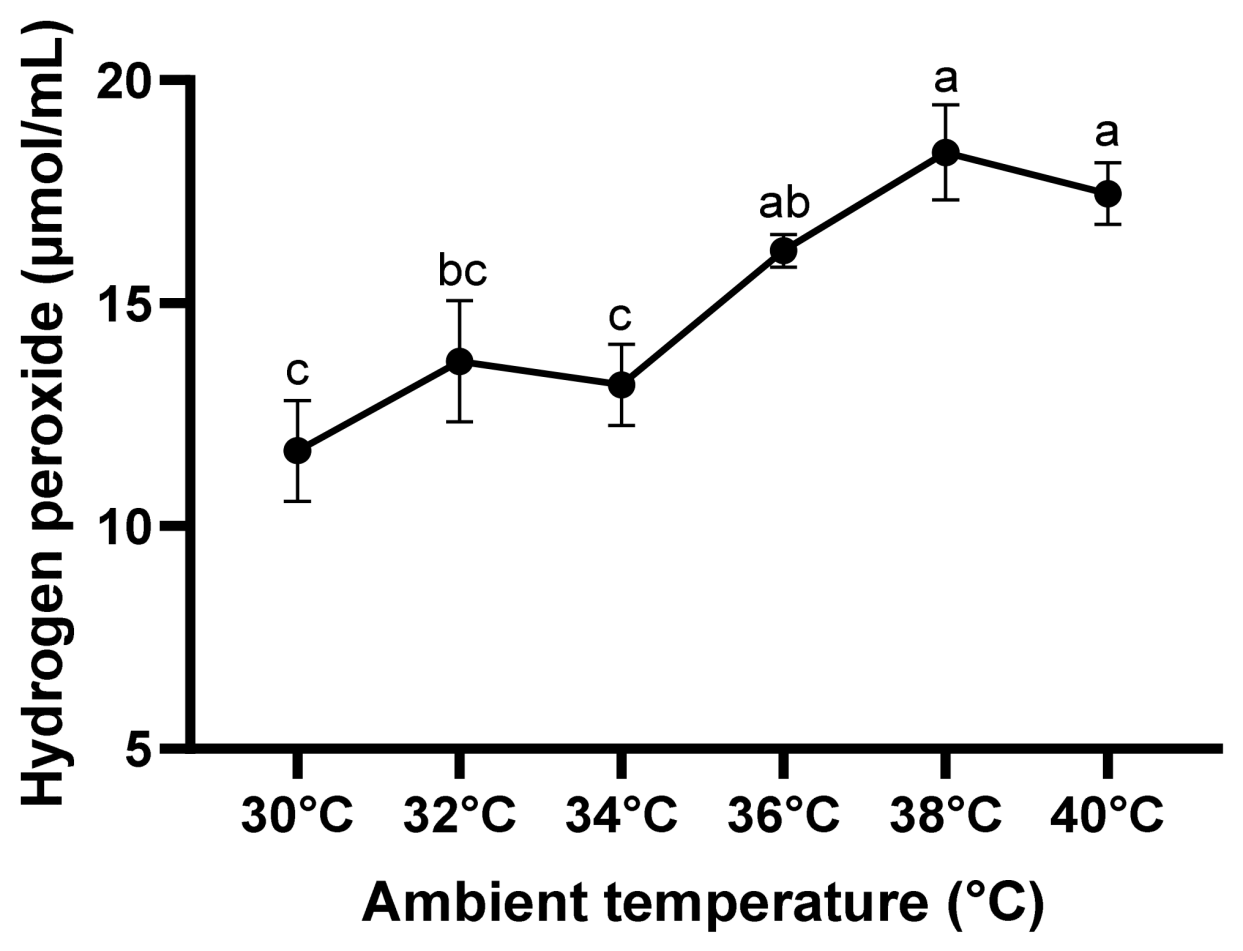
Hydrogen peroxide levels in Siamese crocodile blood cells. Blood from six crocodiles was pooled before temperature exposure to constitute one biological experimental unit. Five technical replicate aliquots were incubated for 20 min at each temperature (30, 32, 34, 36, 38, and 40 °C). Data are presented as mean ± SEM. Different letters indicate significant differences among temperatures (*p* < 0.05). Statistical comparisons and significance letters describe temperature-associated variation among technical replicates within the single pooled preparation and do not represent biological replication or inference at the animal or population level.

**Figure 4:**
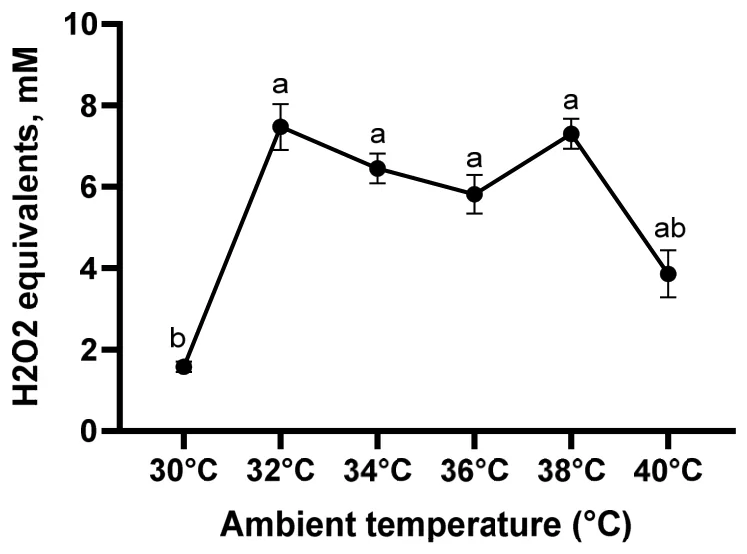
Hydroxyl radical levels, expressed as hydrogen peroxide (H_2_O_2_) equivalents, measured in Siamese crocodile blood cells. Blood from six crocodiles was pooled before temperature exposure to constitute one biological experimental unit. Five technical replicate aliquots were incubated for 20 min at each temperature (30, 32, 34, 36, 38, and 40 °C). Data are presented as mean ± SEM. Different letters indicate significant differences among temperatures (*p* < 0.05). Statistical comparisons and significance letters describe temperature-associated variation among technical replicates within the single pooled preparation and do not represent biological replication or inference at the animal or population level.

**Figure 5:**
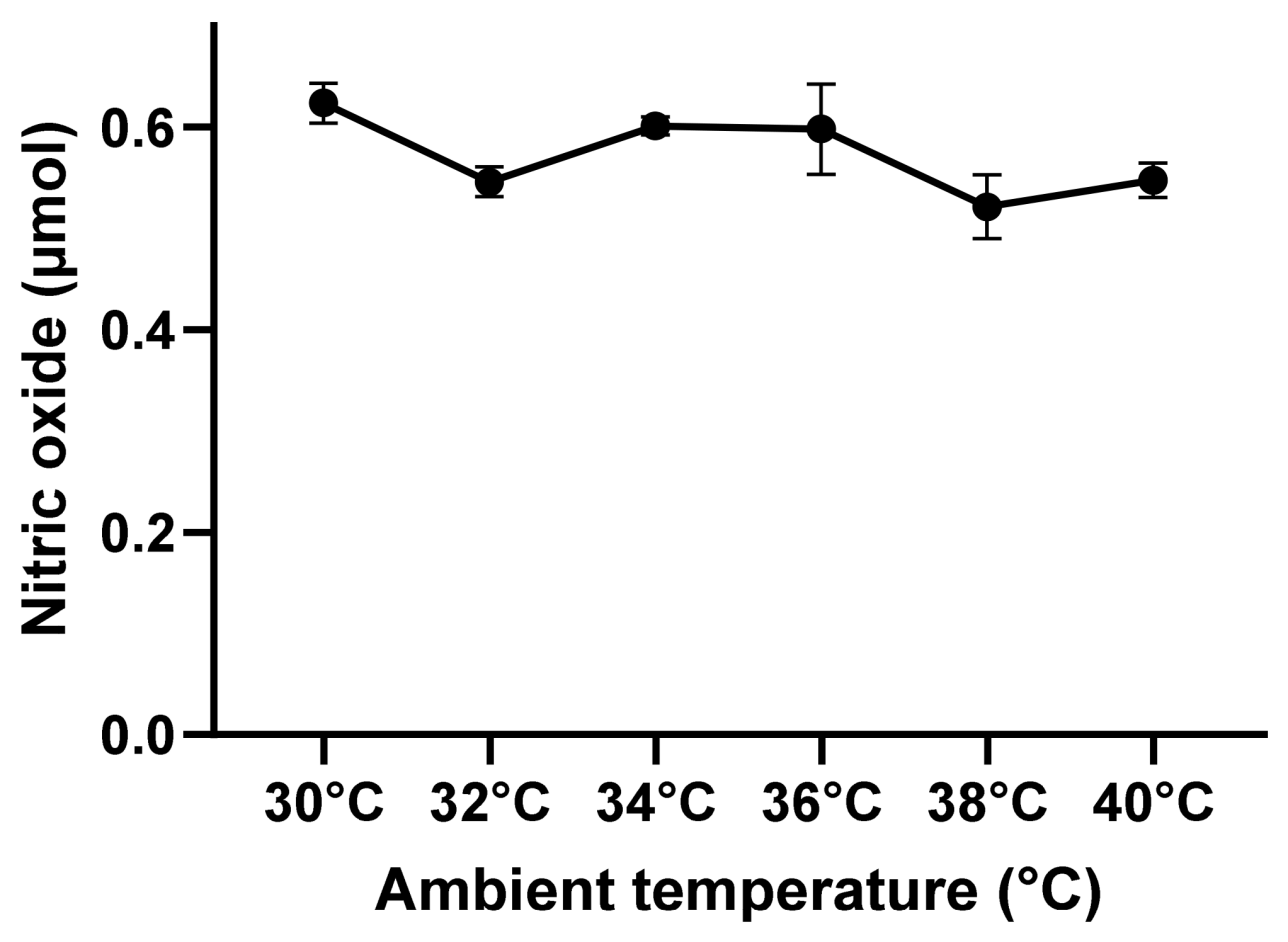
Nitric oxide levels measured in Siamese crocodile blood cells. Blood from six crocodiles was pooled before temperature exposure to constitute one biological experimental unit. Five technical replicate aliquots were incubated for 20 min at each temperature (30, 32, 34, 36, 38, and 40 °C). Data are presented as mean ± SEM. Statistical comparisons describe temperature-associated variation among technical replicates within the single pooled preparation and do not represent biological replication or inference at the animal or population level.

**Figure 6:**
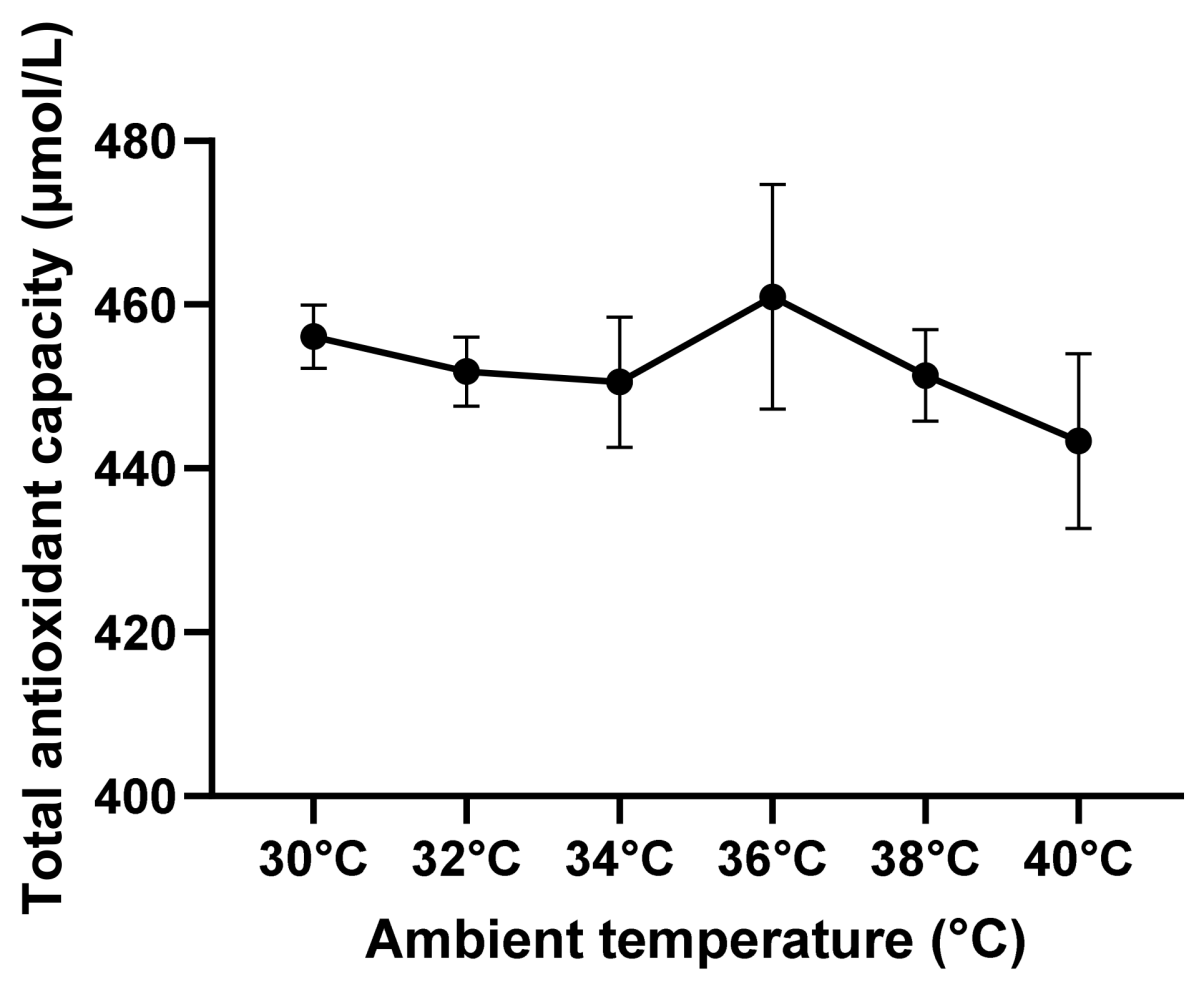
Total antioxidant capacity determined in Siamese crocodile blood cells. Blood from six crocodiles was pooled before temperature exposure to constitute one biological experimental unit. Five technical replicate aliquots were incubated for 20 min at each temperature (30, 32, 34, 36, 38, and 40 °C). Data are presented as mean ± SEM. Statistical comparisons describe temperature-associated variation among technical replicates within the single pooled preparation and do not represent biological replication or inference at the animal or population level.

**Figure 7:**
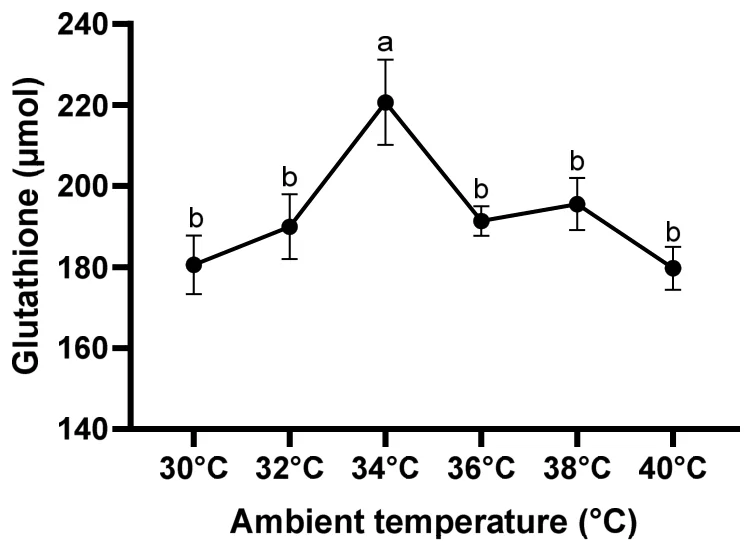
Glutathione levels in Siamese crocodile blood cells. Blood from six crocodiles was pooled before temperature exposure to constitute one biological experimental unit. Five technical replicate aliquots were incubated for 20 min at each temperature (30, 32, 34, 36, 38, and 40 °C). Data are presented as mean ± SEM. Different letters indicate significant differences among temperatures (*p* < 0.05). Statistical comparisons and significance letters describe temperature-associated variation among technical replicates within the single pooled preparation and do not represent biological replication or inference at the animal or population level.

**Figure 8:**
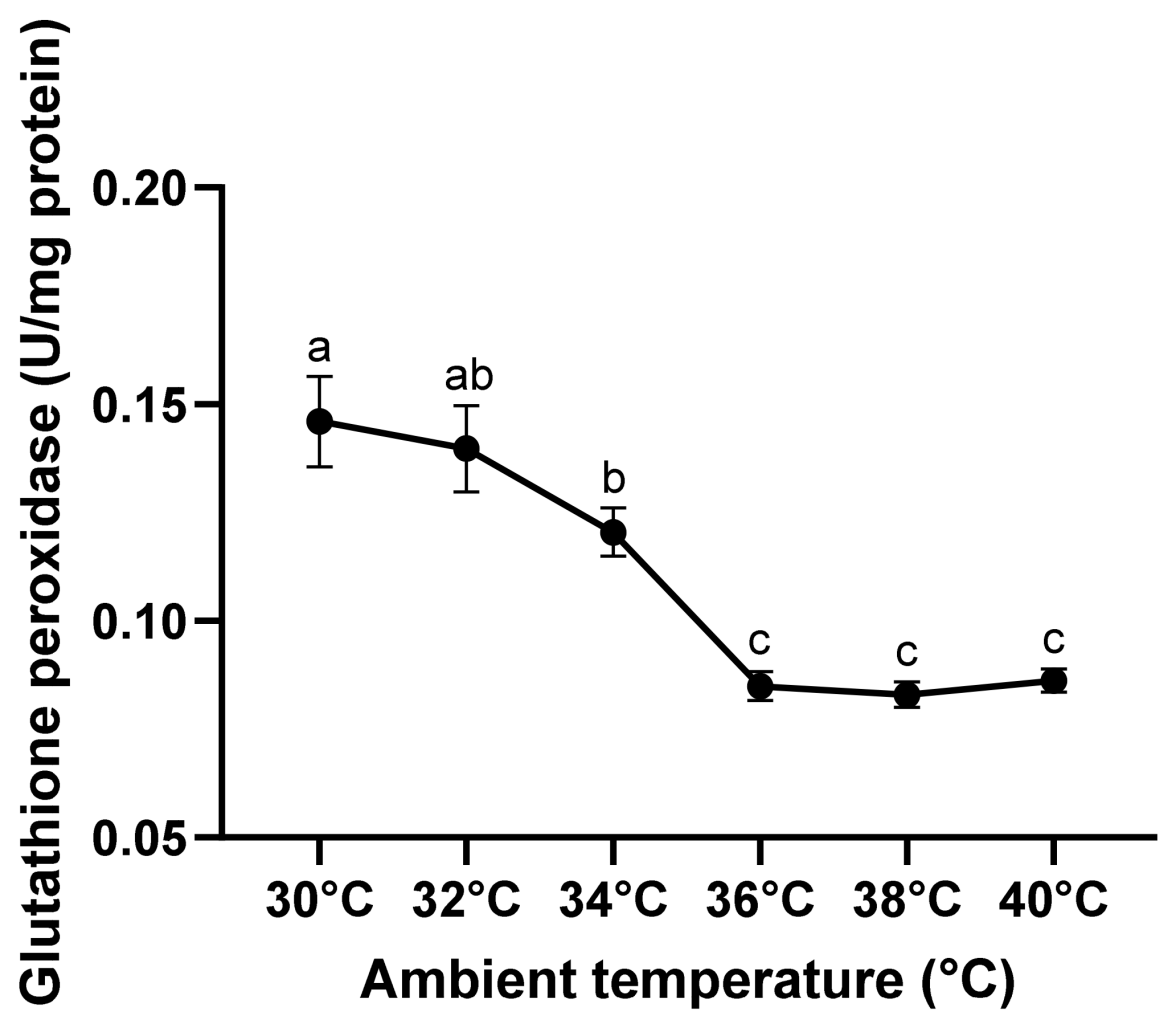
Glutathione peroxidase activity measured in Siamese crocodile blood cells. Blood from six crocodiles was pooled before temperature exposure to constitute one biological experimental unit. Five technical replicate aliquots were incubated for 20 min at each temperature (30, 32, 34, 36, 38, and 40 °C). Data are presented as mean ± SEM. Different letters indicate significant differences among temperatures (*p* < 0.05). Statistical comparisons and significance letters describe temperature-associated variation among technical replicates within the single pooled preparation and do not represent biological replication or inference at the animal or population level.

**Figure 9:**
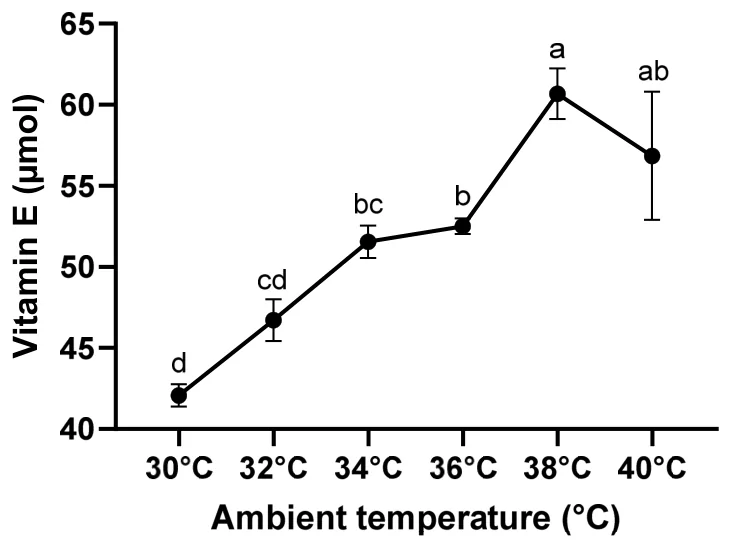
Vitamin E levels determined in Siamese crocodile blood cells. Blood from six crocodiles was pooled before temperature exposure to constitute one biological experimental unit. Five technical replicate aliquots were incubated for 20 min at each temperature (30, 32, 34, 36, 38, and 40 °C). Data are presented as mean ± SEM. Different letters indicate significant differences among temperatures (*p* < 0.05). Statistical comparisons and significance letters describe temperature-associated variation among technical replicates within the single pooled preparation and do not represent biological replication or inference at the animal or population level.

**Figure 10:**
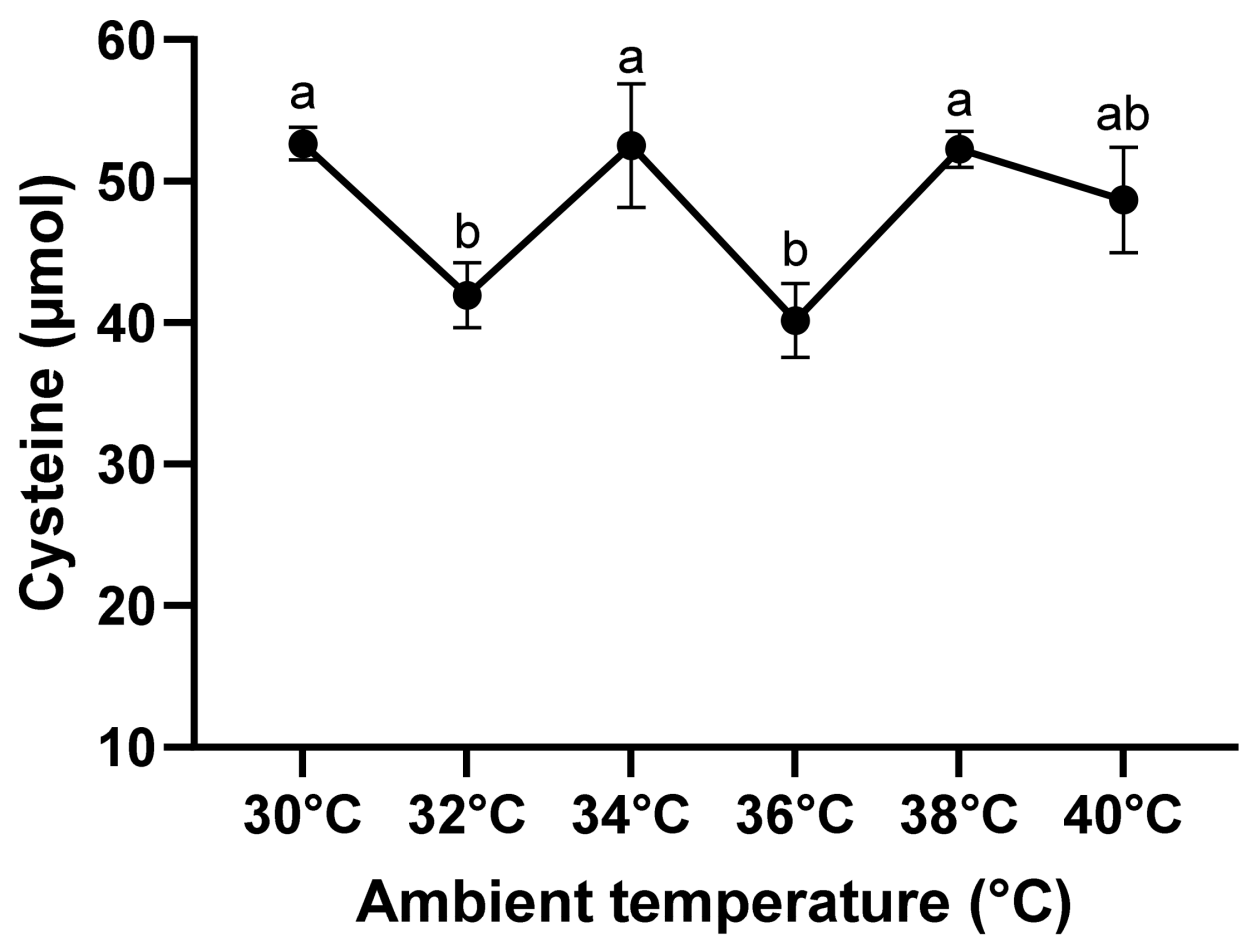
Cysteine levels measured in Siamese crocodile blood cells. Blood from six crocodiles was pooled before temperature exposure to constitute one biological experimental unit. Five technical replicate aliquots were incubated for 20 min at each temperature (30, 32, 34, 36, 38, and 40 °C). Data are presented as mean ± SEM. Different letters indicate significant differences among temperatures (*p* < 0.05). Statistical comparisons and significance letters describe temperature-associated variation among technical replicates within the single pooled preparation and do not represent biological replication or inference at the animal or population level.

**Figure 11:**
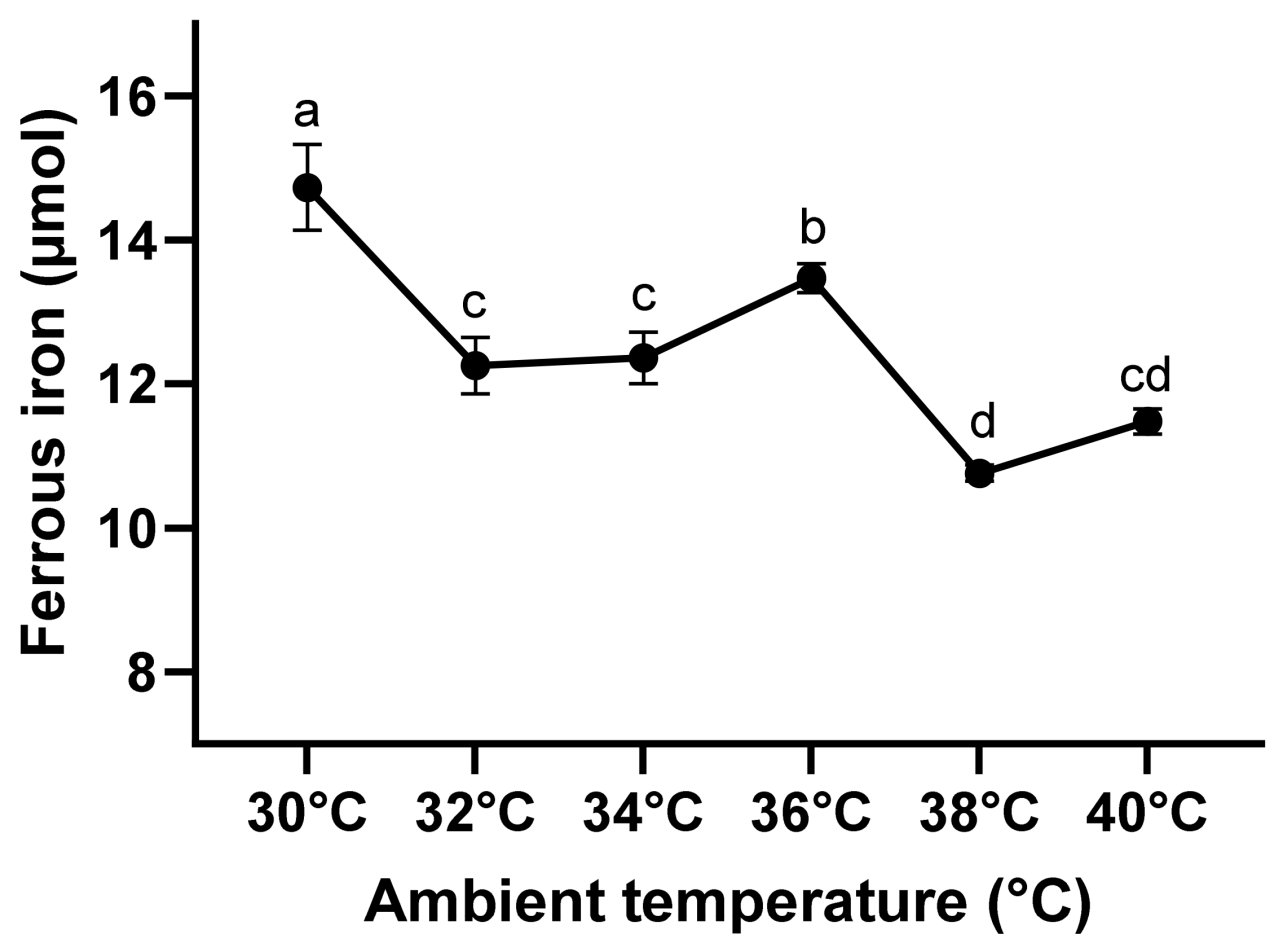
Ferrous iron levels in Siamese crocodile blood cells. Blood from six crocodiles was pooled before temperature exposure to constitute one biological experimental unit. Five technical replicate aliquots were incubated for 20 min at each temperature (30, 32, 34, 36, 38, and 40 °C). Data are presented as mean ± SEM. Different letters indicate significant differences among temperatures (*p* < 0.05). Statistical comparisons and significance letters describe temperature-associated variation among technical replicates within the single pooled preparation and do not represent biological replication or inference at the animal or population level.

**Figure 12:**
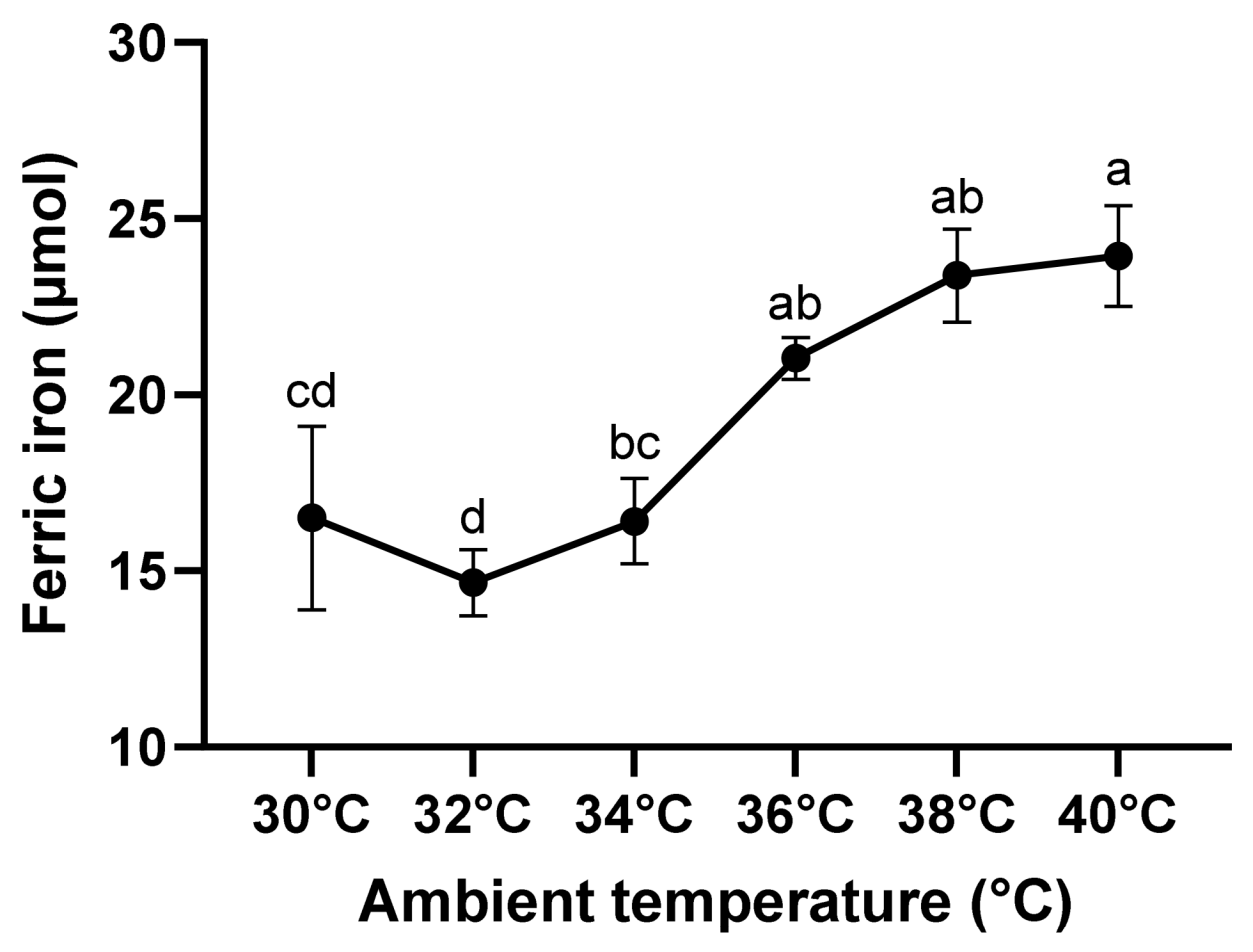
Ferric iron levels determined in Siamese crocodile blood cells. Blood from six crocodiles was pooled before temperature exposure to constitute one biological experimental unit. Five technical replicate aliquots were incubated for 20 min at each temperature (30, 32, 34, 36, 38, and 40 °C). Data are presented as mean ± SEM. Different letters indicate significant differences among temperatures (*p* < 0.05). Statistical comparisons and significance letters describe temperature-associated variation among technical replicates within the single pooled preparation and do not represent biological replication or inference at the animal or population level.

**Figure 13:**
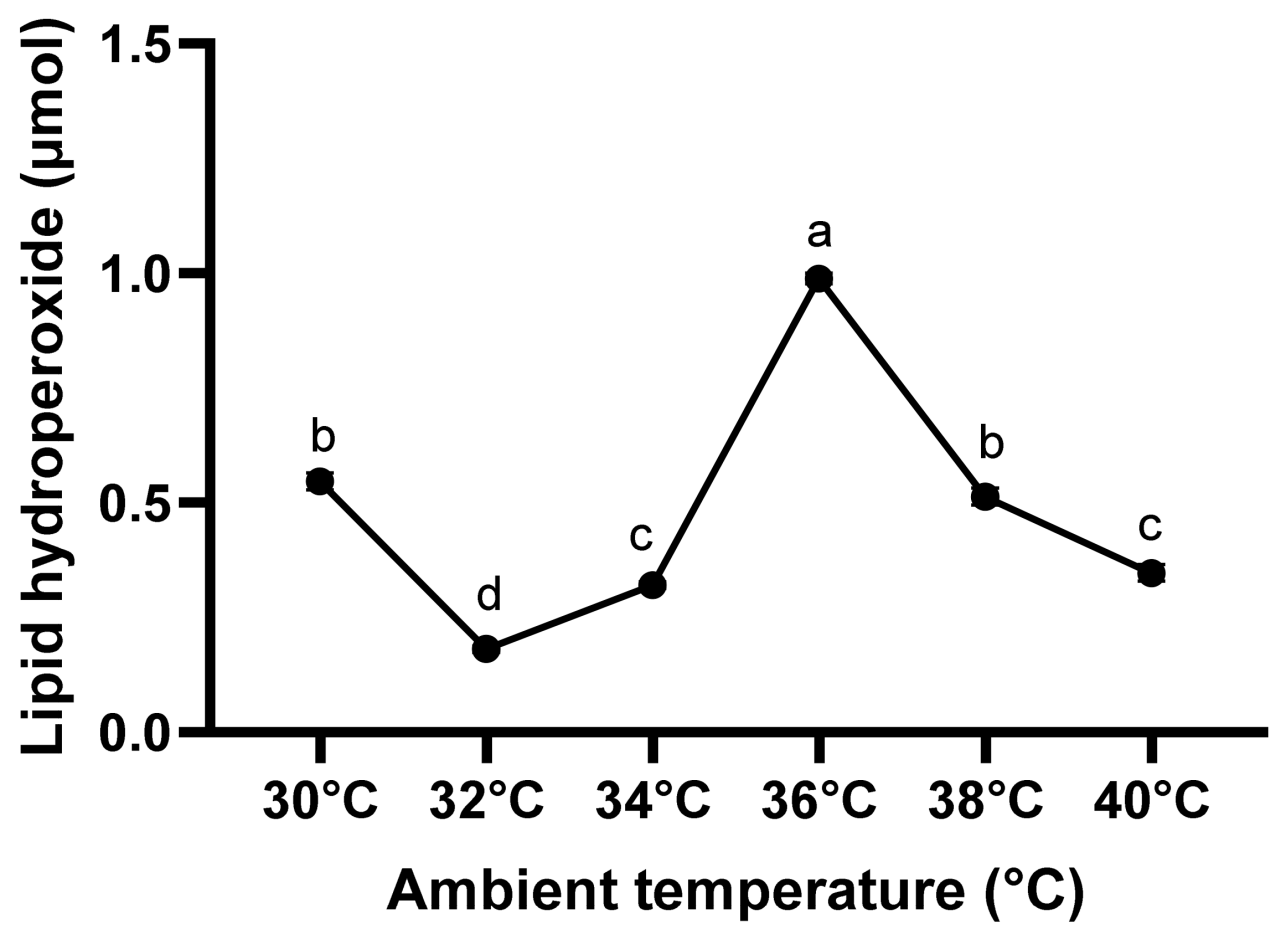
Lipid hydroperoxide levels measured in Siamese crocodile blood cells. Blood from six crocodiles was pooled before temperature exposure to constitute one biological experimental unit. Five technical replicate aliquots were incubated for 20 min at each temperature (30, 32, 34, 36, 38, and 40 °C). Data are presented as mean ± SEM. Different letters indicate significant differences among temperatures (*p* < 0.05). Statistical comparisons and significance letters describe temperature-associated variation among technical replicates within the single pooled preparation and do not represent biological replication or inference at the animal or population level.

**Figure 14:**
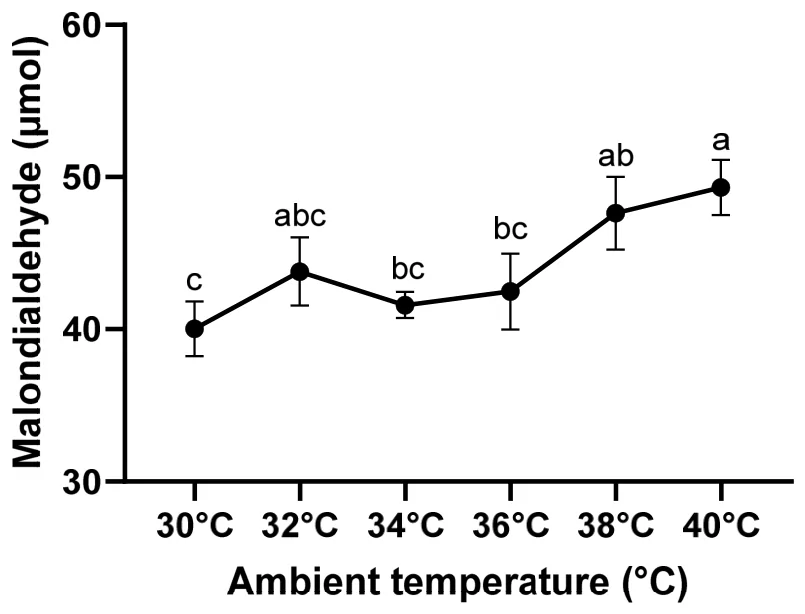
Malondialdehyde levels determined in Siamese crocodile blood cells. Blood from six crocodiles was pooled before temperature exposure to constitute one biological experimental unit. Five technical replicate aliquots were incubated for 20 min at each temperature (30, 32, 34, 36, 38, and 40 °C). Data are presented as mean ± SEM. Different letters indicate significant differences among temperatures (*p* < 0.05). Statistical comparisons and significance letters describe temperature-associated variation among technical replicates within the single pooled preparation and do not represent biological replication or inference at the animal or population level.

**Figure 15:**
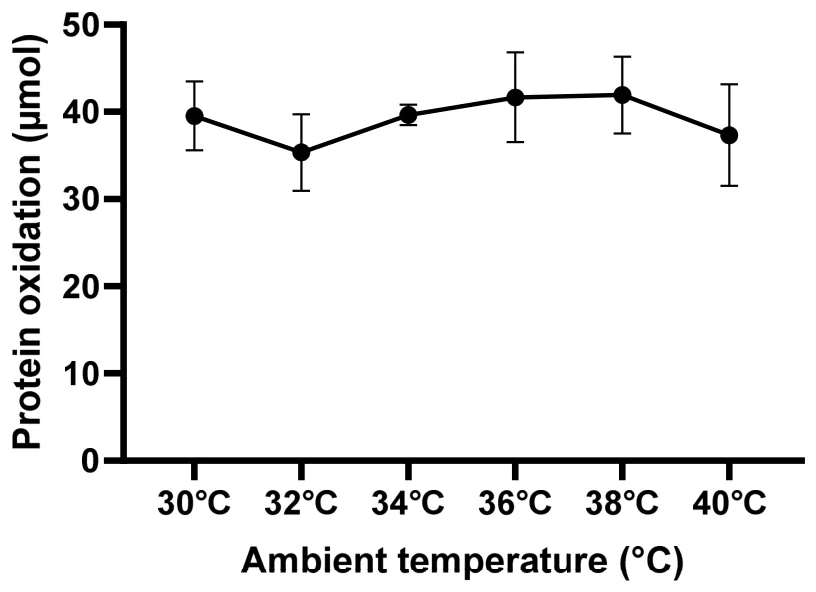
Protein oxidation levels assessed in Siamese crocodile blood cells. Blood from six crocodiles was pooled before temperature exposure to constitute one biological experimental unit. Five technical replicate aliquots were incubated for 20 min at each temperature (30, 32, 34, 36, 38, and 40 °C). Data are presented as mean ± SEM. Statistical comparisons describe temperature-associated variation among technical replicates within the single pooled preparation and do not represent biological replication or inference at the animal or population level.

Figure 2, Figure 3, Figure 4, Figure 5, Figure 6, Figure 7, Figure 8, Figure 9, Figure 10, Figure 11, Figure 12, Figure 13, Figure 14 and Figure 15 present the biochemical findings obtained from the pooled blood-cell preparation. Within this pooled preparation, temperature-associated differences were observed for hydrogen peroxide, hydroxyl radical, glutathione, glutathione peroxidase activity, vitamin E, cysteine, ferrous iron, ferric iron, lipid hydroperoxide, and malondialdehyde (*p* < 0.05). In contrast, mitochondrial activity, nitric oxide, total antioxidant capacity, and protein oxidation showed no statistically detected differences among temperature treatments within the pooled preparation (*p* > 0.05). These biochemical findings represent responses within a single pooled biological preparation, with five technical replicates at each temperature. Detailed pairwise comparisons among temperatures are shown in the corresponding figures.

## 4. Discussion

Physiological responses of Siamese crocodiles during progressive acute warming were examined together with temperature-dependent biochemical responses within a pooled blood-cell preparation. Body temperature is an important physiological indicator in reptiles because these animals are ectothermic and rely on environmental heat sources for thermoregulation [42,43,44]. Body temperature increased progressively as the crocodiles were sequentially exposed to 30 °C, 32 °C, 34 °C, 36 °C, and 38 °C. This pattern is consistent with the dependence of Siamese crocodiles on external thermal conditions for body-temperature regulation. Changes in body temperature may result from heat transfer between the animal and the surrounding environment, together with behavioral mechanisms that regulate heat gain and heat loss [28,42,44]. Previous studies have reported comparable thermal relationships in crocodilians. Grigg et al. [43] found that body temperature in *Crocodylus porosus* varied with environmental temperature and followed a daily thermal cycle, while Loveridge [45] reported increasing body temperature with environmental temperature and basking behavior in Nile crocodiles. Similarly, Seebacher [44] demonstrated that *Crocodylus johnstoni* can modify rates of body-temperature change through posture and body elevation. Therefore, this progressive increase is consistent with the ectothermic thermoregulatory characteristics previously described in crocodilians.

Body surface temperatures also increased during progressive acute warming. Crocodilians rely on environmental heat exchange and behavioral adjustments to regulate body temperature [28,44]. In the present study, surface temperatures of the abdomen, flank, tail base, and thigh were higher at 34–38 °C, while temperatures of the back and claw reached their highest values at 36–38 °C. Head and nose temperatures showed comparatively smaller changes. These regional patterns indicate that surface thermal conditions changed as environmental temperature increased. Previous studies have shown that the extent of water exposure and surrounding thermal conditions can influence rates of body-temperature change in crocodilians [43,44]. Because the crocodiles in the present study remained predominantly immersed in water during thermal exposure, regional differences should be interpreted as surface-temperature patterns under the specific experimental conditions rather than as direct evidence of differences in regional heat-transfer capacity. Nevertheless, surface-temperature measurements may provide a non-invasive indication of changes in body-surface thermal patterns during acute warming.

The increases in body and surface temperatures observed during progressive warming are expected physiological responses in an ectotherm and are therefore not interpreted as novel findings in themselves. Rather, the contribution of the present study lies in integrating these whole-animal responses with temperature-associated changes in reactive oxygen species-related indices, antioxidant defenses, iron redox status, and lipid peroxidation within a standardized pooled blood-cell preparation of *C. siamensis*, for which such integrated information remains limited.

Pulse rate was higher at 36 °C than at 34 °C, whereas no differences were observed across 30–34 °C or between 36 °C and 38 °C. Cardiovascular responses in reptiles can be influenced by body temperature during heating and cooling [28], and temperature-associated changes in heart rate have been reported in *Crocodylus porosus* [28]. However, the pulse rate pattern observed in the present study was nonmonotonic, and the isolated difference between 34 °C and 36 °C does not demonstrate a consistent temperature-dependent cardiovascular response. Because the temperature treatments were applied in a fixed ascending sequence, potential contributions from treatment order, cumulative exposure, repeated handling and restraint, and measurement variability cannot be separated from the effect of increasing temperature. Accordingly, the observed variation in pulse rate should be interpreted cautiously as a response occurring during the progressive acute warming protocol rather than as evidence of a specific cardiovascular mechanism driven by temperature.

The physiological findings should also be considered in relation to the sequential experimental design. All crocodiles were exposed to the same fixed sequence of increasing temperatures. Consequently, increasing temperature was accompanied by increasing cumulative exposure time, treatment order, restraint duration, and repeated handling. Therefore, the physiological changes observed during the experiment cannot be attributed exclusively to temperature independently of these accompanying factors. The present results should thus be interpreted as physiological responses occurring during a controlled progressive acute warming protocol rather than as responses to independent temperature treatments.

In the pooled blood-cell preparation, hydrogen peroxide concentrations were higher at elevated temperatures, particularly from 36 °C onward, with the highest values observed at 38–40 °C. Hydrogen peroxide is formed through cellular oxidative processes, while its removal is mediated by antioxidant enzymes, including catalase and glutathione peroxidase. An imbalance between its generation and removal can contribute to altered redox status [12,46]. Elevated temperature can also influence metabolic and oxidative processes in ectothermic cells [47], although no differences in mitochondrial activity were detected among temperature treatments within the pooled preparation. The increase in hydrogen peroxide occurred concurrently with a decline in glutathione peroxidase (GPx) activity at higher temperatures, consistent with reduced GPx-dependent peroxide-detoxifying capacity. Hydrogen peroxide can also undergo Fenton reactions with ferrous iron, resulting in hydroxyl radical generation, a biochemical process that may contribute to lipid peroxidation [48,49,50]. However, the present study did not directly test causal relationships among these processes.

Hydroxyl radical concentrations increased markedly from 30 °C to 32 °C and remained relatively elevated through 38 °C before declining at 40 °C. This pattern is consistent with temperature-associated changes in highly reactive oxygen species within the pooled blood-cell preparation. Because of their high reactivity, hydroxyl radicals can induce oxidative modification of lipids, proteins, and nucleic acids. They can be generated through Fenton reactions involving hydrogen peroxide and ferrous iron [9,51,52]. The concurrent changes in hydrogen peroxide, ferrous iron, and ferric iron observed in this study are therefore consistent with altered iron-mediated redox processes. Hydroxyl radicals can also initiate lipid peroxidation through reactions with polyunsaturated fatty acids, generating lipid hydroperoxides and other oxidation products [9,52]. This mechanistic context is compatible with the temperature-dependent changes in lipid hydroperoxide and malondialdehyde observed in the pooled preparation.

Glutathione concentrations increased at 34 °C before decreasing at 36–40 °C. Rather than demonstrating a specific adaptive response, the transient elevation at 34 °C may reflect a short-term change in glutathione availability within the pooled blood-cell preparation. Glutathione is an important cellular antioxidant and serves as a reducing substrate for glutathione peroxidase in the reduction of hydrogen peroxide and lipid hydroperoxides [53,54]. The lower glutathione concentrations observed at higher temperatures occurred concurrently with increased oxidative indices and reduced glutathione peroxidase activity. These changes may reflect an altered balance between glutathione availability and its utilization in antioxidant reactions. Impairment of the GSH–GPx antioxidant system can favor lipid peroxide accumulation and is also recognized as one of several biochemical features associated with ferroptosis [52,55].

Glutathione peroxidase activity decreased at higher temperatures and remained relatively low from 36 °C to 40 °C. This pattern is consistent with reduced GPx-dependent peroxide-detoxifying capacity within the pooled blood-cell preparation and occurred concurrently with changes in hydrogen peroxide, lipid hydroperoxide, and malondialdehyde. Cellular redox regulation is supported by glutathione peroxidase, which uses glutathione as a reducing substrate to remove hydrogen peroxide and lipid hydroperoxides [12,56]. Reduced glutathione peroxidase activity may favor peroxide accumulation when oxidative processes exceed antioxidant capacity, potentially contributing to lipid peroxidation [9,57].

Vitamin E concentrations generally increased at higher temperatures and reached relatively high levels at 38–40 °C. As a lipid-soluble antioxidant, vitamin E helps preserve membrane lipids by neutralizing lipid peroxyl radicals and terminating the chain propagation of lipid peroxidation [12,58]. Therefore, the higher vitamin E concentrations observed at elevated temperatures may be consistent with changes in lipid-phase antioxidant status during increased oxidative challenge [59]. In contrast, total antioxidant capacity remained unchanged despite the reduction in glutathione peroxidase activity. This pattern may reflect contributions from multiple antioxidant components, including non-enzymatic antioxidants; however, the present experiment did not directly determine the relative contributions or compensatory activity of individual antioxidant systems [12,60].

Cysteine concentrations varied among temperatures without a simple progressive pattern. Cysteine is a sulfur-containing amino acid and an important precursor for glutathione synthesis, thereby contributing to redox regulation and antioxidant defense [61,62]. The observed fluctuations may therefore reflect temperature-associated variation in cysteine availability within the pooled blood-cell preparation. Changes in cysteine may be related to its involvement in glutathione metabolism and thiol redox regulation, although these processes were not directly assessed under the experimental conditions [61,63].

Ferrous iron concentrations varied among temperatures, with the lowest value observed at 38 °C. These changes occurred concurrently with higher hydrogen peroxide and malondialdehyde concentrations at elevated temperatures and are consistent with altered iron redox status during thermal exposure. During the Fenton reaction, hydrogen peroxide oxidizes redox-active ferrous iron to its ferric form, accompanied by the formation of hydroxyl radicals. This reaction provides a mechanistic link between iron redox cycling, reactive oxygen species production, and oxidative modification of lipids [64,65,66]. Thus, the concurrent changes in ferrous iron, hydrogen peroxide, hydroxyl radicals, and lipid-peroxidation indices are consistent with the possibility of increased iron-associated oxidative reactions at higher temperatures.

Ferric iron concentrations were higher at 36–40 °C than at lower temperatures, indicating a temperature-associated shift in iron redox status within the pooled blood-cell preparation. Ferrous iron can be oxidized to ferric iron during Fenton chemistry, while ferric iron may subsequently be reduced to ferrous iron by cellular reducing systems, thereby sustaining iron redox cycling [67,68]. Therefore, the higher ferric iron concentrations, together with temperature-dependent changes in ferrous iron and hydrogen peroxide, are compatible with altered iron-mediated oxidative processes under the experimental conditions. Such redox processes can contribute to lipid peroxidation, particularly in membranes containing polyunsaturated fatty acids [69,70,71].

Lipid hydroperoxide concentrations peaked at 36 °C and subsequently decreased at 38–40 °C. This pattern suggests that the balance among lipid-peroxide formation, conversion, and removal varied across temperatures. Lipid hydroperoxides are primary products of lipid peroxidation and may subsequently be converted into secondary oxidation products, including aldehydes such as malondialdehyde [72,73]. The peak in lipid hydroperoxide at 36 °C occurred together with reduced glutathione peroxidase activity, which could contribute to greater lipid hydroperoxide accumulation because the GSH–GPx system participates in lipid-peroxide detoxification [53,73]. Conversely, the decline at 38–40 °C may reflect further conversion to secondary lipid-peroxidation products or changes in lipid-phase antioxidant activity, including the action of α-tocopherol [74]. These possibilities remain mechanistic interpretations rather than processes directly demonstrated in the present experiment.

Malondialdehyde concentrations were higher at the upper experimental temperatures, particularly at 38–40 °C, supporting increased lipid-peroxidation-related changes in the pooled blood-cell preparation. Malondialdehyde is a secondary product of lipid peroxidation and serves as a marker of oxidative damage to membrane lipids [75]. Oxidative attack by reactive oxygen species on polyunsaturated fatty acids can lead to the formation of lipid hydroperoxides and secondary aldehydes, including malondialdehyde [9,75]. Iron-dependent Fenton chemistry may further contribute to these reactions through hydroxyl radical generation [48,76]. Therefore, the concurrent temperature-associated changes in malondialdehyde, hydrogen peroxide, and iron redox status are consistent with an association between altered oxidative and iron redox conditions and lipid peroxidation within the pooled preparation.

Several biochemical indices exhibited nonmonotonic responses to increasing temperature, indicating that the measured concentrations likely reflect the net balance of multiple concurrent processes rather than a simple temperature-dependent dose-response relationship. These processes may include reactive oxygen species generation and removal, antioxidant activity, iron redox cycling, and the formation, decomposition, and further transformation of lipid peroxidation products. For example, lipid hydroperoxide reached its highest level at 36 °C and subsequently decreased at 38–40 °C, whereas malondialdehyde was higher at the upper temperatures. This pattern may reflect changes in the balance among lipid hydroperoxide formation, detoxification, decomposition, and further transformation, although the present study does not establish a direct conversion pathway between these endpoints. Similarly, the nonmonotonic hydroxyl radical pattern may reflect a changing balance between radical generation and removal together with temperature-associated changes in iron redox reactions. These interpretations are therefore considered plausible biochemical explanations rather than mechanisms directly demonstrated by the present study.

**Figure 16:**
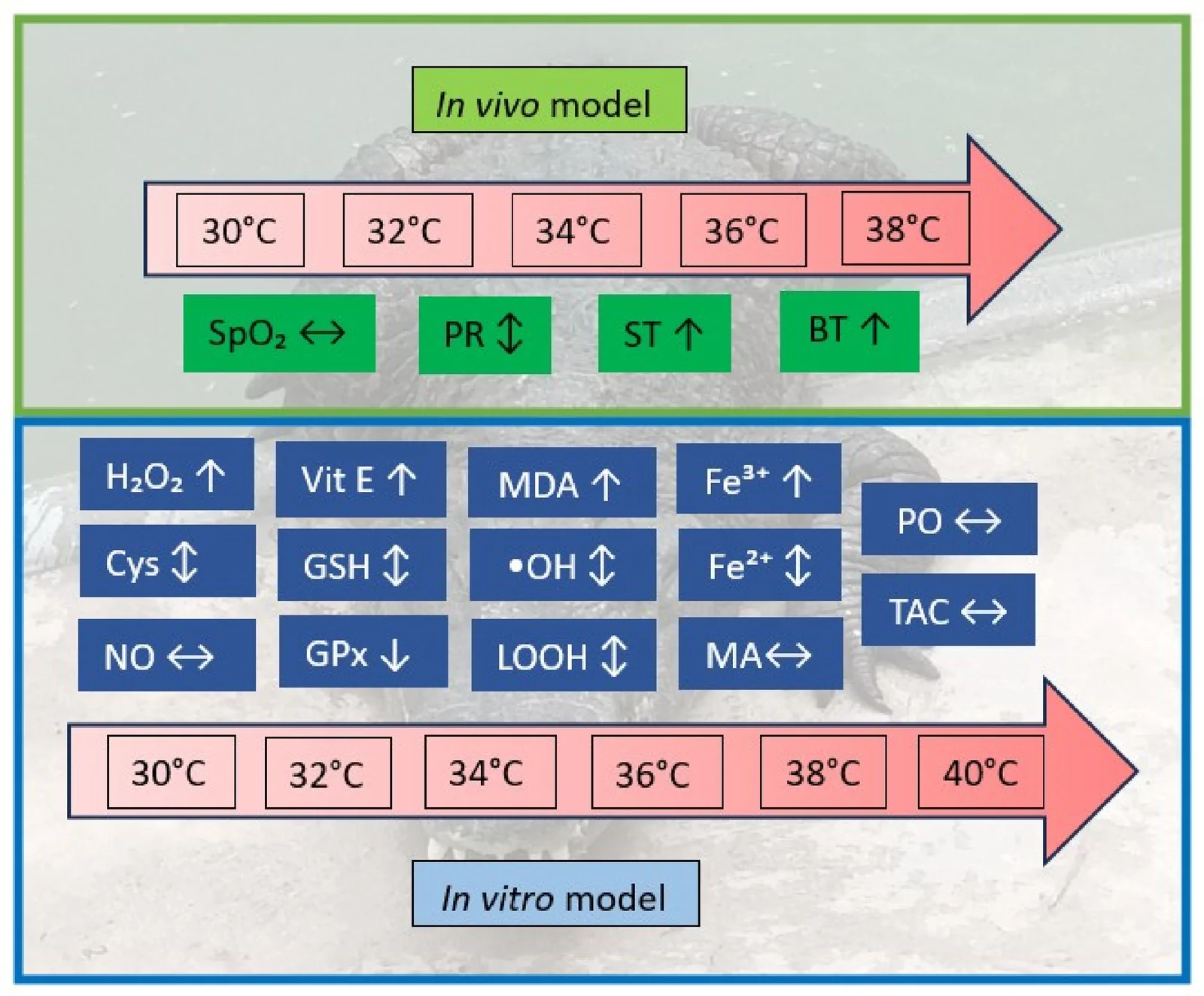
Integrated overview of physiological and biochemical responses to acute temperature elevation in Siamese crocodiles. In the in vivo experiment, crocodiles were sequentially exposed to water temperatures of 30, 32, 34, 36, and 38 °C, and physiological responses were assessed. In the in vitro experiment, pooled blood cells were exposed to 30, 32, 34, 36, 38, and 40 °C for 20 min, and biochemical responses were evaluated. Arrows indicate the overall response patterns across the experimental temperatures: ↑, increased; ↓, decreased; ↕, variable or nonmonotonic response; and ↔, no statistically detected difference among temperature treatments. Abbreviations: BT, body temperature; ST, surface temperature; PR, pulse rate; SpO_2_, blood oxygen saturation; H_2_O_2_, hydrogen peroxide; •OH, hydroxyl radical; NO, nitric oxide; TAC, total antioxidant capacity; GSH, glutathione; GPx, glutathione peroxidase; Vit E, vitamin E; Cys, cysteine; Fe^2+^, ferrous iron; Fe^3+^, ferric iron; LOOH, lipid hydroperoxide; MDA, malondialdehyde; PO, protein oxidation; MA, mitochondrial activity. Biochemical response patterns represent temperature-associated changes within a single pooled blood-cell preparation and should not be interpreted as animal-level or population-level responses.

Overall, progressive acute warming was accompanied by physiological changes in Siamese crocodiles, while the pooled in vitro blood-cell preparation exhibited temperature-dependent biochemical alterations. Several changes became apparent from approximately 36 °C onward under the present experimental conditions. Physiologically, body and surface temperatures increased during progressive warming, and pulse rate differed between selected temperatures, whereas blood oxygen saturation remained unchanged. However, unchanged blood oxygen saturation alone does not establish whether tissue oxygen supply fully met metabolic demand. At the biochemical level, higher temperatures were associated with changes in hydrogen peroxide, the GSH–GPx antioxidant system, vitamin E, iron redox status, and lipid-peroxidation indices. Collectively, these responses indicate altered oxidative and antioxidant conditions within the standardized pooled blood-cell preparation (Figure 16).

The concurrent changes in reactive oxygen species-related indices, antioxidant defenses, iron redox status, and lipid peroxidation share several biochemical features associated with ferroptosis, particularly alterations in the GSH–GPx antioxidant system, iron-dependent redox reactions, and lipid peroxide accumulation. However, ferroptotic cell death and ferroptosis-specific molecular pathways were not examined under the experimental conditions. Accordingly, the observed biochemical patterns may be considered potentially relevant to ferroptosis but do not demonstrate that ferroptosis occurred in Siamese crocodile blood cells.

Several physiological and biochemical changes became apparent from approximately 36 °C onward under the present experimental conditions. However, 36 °C was neither defined a priori as a thermal threshold nor derived from breakpoint, change point, or other threshold analysis. It should therefore be interpreted only as a descriptive experimental temperature around which several measured responses showed concurrent alterations. The present findings do not establish 36 °C as a thermal tolerance, welfare, or husbandry threshold. Studies specifically designed to determine thermal tolerance limits, incorporating independent biological replication and appropriate threshold analyses, are required before management recommendations can be established.

Finally, the findings need to be interpreted in light of several study limitations. First, the in vivo thermal exposures followed a fixed progressive sequence; therefore, temperature could not be completely separated from cumulative exposure time, treatment order, restraint duration, and repeated handling. Second, blood from all six crocodiles was pooled before the in vitro experiment. Consequently, the pooled blood-cell preparation constituted a single biological experimental unit, while the five aliquots at each temperature represented technical replicates. Statistical differences among temperatures therefore describe variation within this standardized pooled preparation and should not be interpreted as population-level inference among individual crocodiles. Moreover, biochemical responses observed in the pooled blood-cell preparation cannot be assumed to reproduce tissue- or organ-specific responses or whole-animal physiological responses and should therefore be interpreted as complementary cellular findings under the present experimental conditions. Third, several concentration-based biochemical indices were measured in the experimental preparation without normalization to cell number or protein content. Temperature-associated alterations in membrane integrity and subsequent cellular release or leakage may therefore have contributed to the measured concentrations. Because membrane integrity and cellular leakage were not directly assessed, the present data cannot distinguish changes arising from altered intracellular production or metabolism from those resulting from cellular release or leakage, nor can they determine the relative contribution of these mechanisms to the observed biochemical patterns. Fourth, the study included three male and three female crocodiles but was not designed or statistically powered to evaluate sex-specific responses; consequently, possible sex-related variation cannot be excluded. Despite these limitations, the study provides a controlled characterization of short-term physiological responses during progressive warming and complementary temperature-dependent biochemical patterns within a standardized Siamese crocodile blood-cell preparation.

## 5. Conclusions

Progressive acute warming was accompanied by increases in body and surface temperatures and variation in pulse rate in Siamese crocodiles, whereas blood oxygen saturation remained unchanged. Within the pooled blood-cell preparation, increasing temperature was associated with alterations in oxidative balance, antioxidant defenses, iron redox status, and lipid peroxidation indices, with several changes becoming apparent from approximately 36 °C onward. These biochemical patterns involve processes that are also associated with ferroptosis; however, ferroptotic cell death and ferroptosis-specific molecular pathways were not directly investigated. Overall, the findings provide a controlled characterization of short-term physiological and biochemical responses to acute temperature elevation in Siamese crocodiles. Approximately 36 °C should be regarded only as a descriptive experimental observation and not as a statistically defined thermal tolerance, welfare, or husbandry threshold.

## Figures and Tables

**Table 1:** Physiological responses of Siamese crocodiles during progressive water warming from 30 °C to 38 °C, including body temperature, regional surface temperatures, pulse rate, and blood oxygen saturation (*n* = 6 biological replicates).

Parameters	Ambient Temperature (°C)					*p*-Value
	30 °C	32 °C	34 °C	36 °C	38 °C	
1. Body temperature (°C)	31.35 ± 0.15 ^e^	32.53 ± 0.15 ^d^	33.92 ± 0.33 ^c^	36.33 ± 0.14 ^b^	37.48 ± 0.20 ^a^	<0.0001
2. Surface temperature (°C)						
2.1 Abdominal temperature	28.03 ± 0.20 ^c^	28.77 ± 0.20 ^c^	31.15 ± 0.41 ^b^	32.30 ± 0.30 ^ab^	33.04 ± 0.63 ^a^	<0.0001
2.2 Back temperature	28.95 ± 0.73 ^bc^	27.75 ± 0.73 ^c^	30.63 ± 0.62 ^ab^	32.18 ± 0.80 ^a^	32.93 ± 0.66 ^a^	<0.0001
2.3 Claw temperature	27.95 ± 0.25 ^c^	28.57 ± 0.25 ^bc^	30.70 ± 0.32 ^ab^	32.28 ± 0.54 ^a^	32.23 ± 0.54 ^a^	<0.0001
2.4 Flank temperature	27.97 ± 0.34 ^c^	27.83 ± 0.34 ^c^	31.02 ± 0.59 ^b^	32.18 ± 0.39 ^ab^	33.03 ± 0.25 ^a^	<0.0001
2.5 Head temperature	31.12 ± 1.29 ^ab^	28.85 ± 1.29 ^b^	31.55 ± 0.97 ^ab^	31.95 ± 1.60 ^a^	34.13 ± 0.69 ^a^	0.0262
2.6 Nose temperature	28.57 ± 0.62 ^ab^	28.02 ± 0.62 ^b^	29.22 ± 0.43 ^ab^	30.05 ± 0.82 ^ab^	30.65 ± 0.46 ^a^	0.0107
2.7 Tail base temperature	27.78 ± 0.45 ^c^	28.08 ± 0.45 ^c^	30.97 ± 0.43 ^b^	31.78 ± 0.27 ^ab^	32.60 ± 0.31 ^a^	<0.0001
2.8 Thigh temperature	27.88 ± 0.43 ^c^	27.97 ± 0.43 ^c^	30.43 ± 0.67 ^ab^	31.78 ± 0.27 ^ab^	32.77 ± 0.26 ^a^	<0.0001
3. Pulse rate (bpm)	58.38 ± 6.86 ^ab^	49.16 ± 6.86 ^ab^	42.50 ± 5.82 ^b^	62.38 ± 6.33 ^a^	55.03 ± 6.33 ^ab^	0.0360
4. Oxygen saturation (%)	100.00 ± 0.00	99.17 ± 0.65	100.00 ± 0.00	99.67 ± 0.33	99.83 ± 0.17	0.3714

Within each row, significant differences among temperatures are indicated by different letters (*p* < 0.05). Data are presented as mean ± SEM.

## Data Availability

The original contributions presented in this study are included in the article. Further inquiries can be directed to the corresponding author.
